# Oscillospira - a candidate for the next-generation probiotics

**DOI:** 10.1080/19490976.2021.1987783

**Published:** 2021-10-25

**Authors:** Jingpeng Yang, Yanan Li, Zhiqiang Wen, Wenzheng Liu, Lingtong Meng, He Huang

**Affiliations:** School of Food Science and Pharmaceutical Engineering, Nanjing Normal University, Nanjing, China

**Keywords:** *Oscillospira*, obesity, human health, short-chain fatty acids (scfas), next-generation probiotics

## Abstract

*Oscillospira* is a class of organism that often appears in high-throughput sequencing data but has not been purely cultured and is widely present in the animal and human intestines. There is a strong association between variation in *Oscillospira* abundance and obesity, leanness, and human health. In addition, a growing body of studies has shown that *Oscillospira* is also implicated in other diseases, such as gallstones and chronic constipation, and has shown some correlation with the positive or negative changes in its course. Sequencing data combined with metabolic profiling indicate that *Oscillospira* is likely to be a genus capable of producing short-chain fatty acids (SCFAs) such as butyrate, which is an important reference indicator for screening “next-generation probiotics “. Considering the positive effects of *Oscillospira* in some specific diseases, such as obesity-related metabolic diseases, it has already been characterized as one of the next-generation probiotic candidates and therefore has great potential for development and application in the future food, health care, and biopharmaceutical products.

## 1. Introduction

In the past two decades, with the continuous development of high-throughput sequencing technology, people have gained further understanding of intestinal microorganisms, and the accuracy of species resolution is also getting higher, and some mysterious “dark matter” harbored in the gut is being revealed step by step^1^. From 16S rRNA amplicon sequencing to the widespread use of metagenomic technologies, growing data show that *Oscillospira* frequently appears in various gut microbiota-related studies, but so far, this organism has never been purely cultured and its metabolic and biological characteristics are still unraveled. Several studies have shown a significant positive association between *Oscillospira* and low fat, leanness, and human health,^2^ in addition to a strong association between this organism and a variety of diseases, especially inflammatory diseases.^2,3^ Gut and fecal microbiome sequencing data show that *Oscillospira* accounts for a high proportion of the human fecal microbiome, suggesting that this organism is likely to play an important role in human health.^4^ Meanwhile, several studies have shown that *Oscillospira* can produce all kinds of short-chain fatty acids (SCFAs) dominated by butyrate.^2,3,5^ Therefore, whether *Oscillospira* has potential for development as probiotics and whether it can be the most favorable next-generation probiotic candidate on par with *Faecalibacterium prausnitzii, Akkermansia muciniphila*, and *Eubacterium hallii* deserves further research.^6^

### 1.1. *Know features of* oscillospira

Records of *Oscillospira* first appeared a century ago when Chatton and Pérard discovered *Oscillospira guilliermondii* in guinea pig cecal contents, which is the only known species within the genus *Oscillospira*.^7^ However, little is known about its ecological role and physiological properties in the intestine because pure cultures have not been obtained.^8^ Since *O. guilliermondii* is large and morphologically distinct, which facilitates its DNA isolation and 16S rRNA gene amplification and sequencing, the genus *Oscillospira* was classified based on flow cytometry and determined by 16S rRNA phylogenetic analysis to be a member of the family *Ruminococcaceae*, order *Clostridiales*, class *Clostridia* in the phylum Firmicutes.^7,8^ Some members of the genus *Oscillospira* are generally rod-shaped or ellipsoidal, 3–6 μm in diameter and 10–40 μm in length, especially *O. guilliermondii* cells are very large (about 5–7 μm in width, and up to 70 mm in length), and intracellularly closely spaced transverse septa could be seen under transmission electron microscopy.^7^ Some of *Oscillospira* have endospores (2.5 × 4 μm), arranged longitudinally in rods, refractile and variable in number, which usually contain a large number of polysaccharides and appear reddish or mauve in the presence of iodine.^7^ Since spore-like structures were observed in *Oscillospira*, it is speculated that this group of microorganisms may contain spore-associated genes.^9^ Gene-level analysis revealed the presence of the small acid-soluble spore protein, spore maturation proteins A and B, six stage III sporulation proteins, and the sporulation transcriptional regulators SpoIIID, and SpoVT in some members of *Oscillospira*, however, some have no SpoVT and other related spore proteins,^3^ suggesting that sporulation is likely a sporadically distributed feature in the *Oscillibacter* clade. Interestingly, sporulation genes are also present in some non-sporulating species, such as *Oscillibacter valericigenes*, which contain sporulation genes but do not produce spores, and these sporulation genes may play other roles, such as regulatory genes.^10^ *Oscillospira* is a Gram-positive bacteria with low G + C content, whose sequence is close to that of uncultured bacterial clones within the clostridial cluster IV (Collins nomenclature) obtained from the cecum of broiler chickens and rumen contents of dairy cows.^8^ Interestingly, clonal sequences from human fecal samples also belong to this cluster, suggesting that microorganisms within this cluster are widespread not only in the digestive tract of herbivores, but also in the digestive tract of omnivores.^7^

Starting from a metagenomic and metabolic signature perspective, Gophna et al. used sequence similarity, gene neighborhood information, and artificial metabolic pathway screening to decipher key features of *Oscillospira* and found that this organism has a butyrate kinase-mediated pathway, from which it was inferred that *Oscillospira* is a butyrate producer and that at least some of these species can utilize gluconate, a common animal-derived sugar that is both produced by the human host and ingested by the host through a diet rich in animal products.^3^ On the other hand, it was further demonstrated that *Oscillospira* can ferment complex plant carbohydrates.^11^ Specific carbon sources are also essential for its growth, e.g. *Oscillospira* grows well in media containing glucose, ethanol, and lactic acid, and glucose in particular significantly promotes its growth.^12^ It has been shown that *Oscillospira* is difficult to culture and slow to grow, which may be related to the longer colonic transit time.^3^ Fast colonic transit times select for fast-growing microbes, and by the same token, slower transit conditions allow slower microbes to remain in the lumen and avoid being eluted,^13^ a property that the slow-growing *Oscillospira* seems to fit. On the other hand, the number of tRNA genes in the genome can serve as a strong predictor when microbial generations.^14^ The vast majority of fast-growing microbes have more copies of tRNA genes in their genomes, and vice versa. Previous studies have shown that there are less than 40 tRNA genes in *Oscillospira*, and compared to other fast-growing intestinal microbes such as *Bacteroides fragilis* (72–73 tRNA genes, generation time 0.63 h), *Clostridioides difficile* (82 tRNA genes, generation time of < 70 minutes), *Oscillospira* is typically a very slow-growing organism.^3^ In recent years, multiple 16S rRNA amplicon sequencing data based on the human gut microbiome have shown that *Oscillospira* is an abundant component of the human gut and fecal microbiota, and its gene sequence amount sometimes account for more than 10% of the entire gut microbiota,^4^ meanwhile, *Oscillospira* can produce butyrate,^3^ suggesting that this organism may play an important role in various aspects of human bodily functions and health, therefore, more research data are needed to reveal its properties.

## 2. Factors affecting the abundance of *oscillospira*

**Figure 1.**
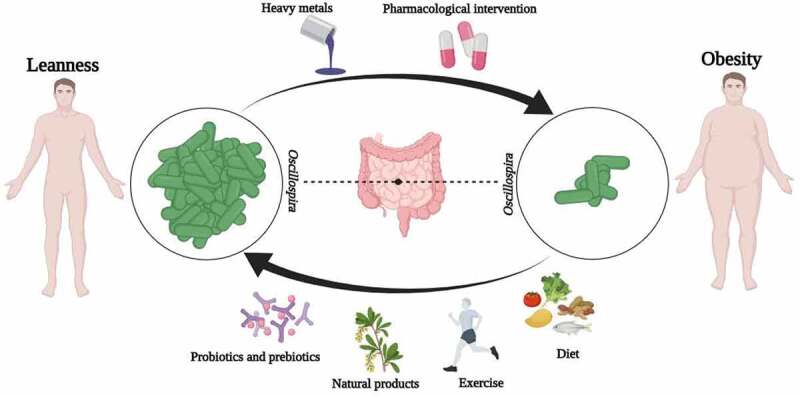
Various factors are affecting *oscillospira* abundance in the human gut. probiotics, prebiotics, natural products, exercise, and diet can positively regulate *oscillospira* abundance, whereas heavy metals and pharmacological interventions can negatively regulate *oscillospira* abundance in the gut

Various factors are affecting *Oscillospira* abundance, mainly exogenous factors (Table 1). It is known from a range of literature that probiotics, prebiotics, heavy metals, natural active products, pharmacological interventions, exercise and diet, and other factors can have an impact on the abundance of *Oscillospira* in the gut (Figure 1).

**Table 1.** Factors affecting the abundance of *oscillospira.*

Category	Factors	Positive (+)/Negative (-)	references
Probiotics and prebiotics	*Bacillus coagulans* 13002	**+**	^15^
	*Bacillus subtilis* 29,784	**+**	^16^
	*Bacillus amyloliquefaciens* & *Bacillus subtilis*	**+**	^17^
	*Bifidobacterium breve* ATCC 15700	**+**	^18^
	*Leuconostoc pseudomesenteroides* XG5-exopolysaccharide	**+**	^19^
	Tibet kefir milk (TKM)	**+**	^20^
	*Lactobacillus rhamnosus*	-	^21^
	*Clostridium butyricum*	-	^22^
	Fructo-oligosaccharides (FOS)	**+**	^15^
	Fucoidan (FUC)	**+**	^23^
	Galactooligosaccharides (GOS)	**+**	^23^
	Fucosyl-α1,3-GlcNAc (3FN); Fucosyl-α1,6-GlcNAc; Lacto-N-biose (LNB); Galacto-N-biose	**+**	^24^
	Pea fiber	**+**	^25^
	Potato fiber (FiberBind 400)	**+**	^26^
	Oat β-glucan (OG); Oat resistant starch (ORS); Whole oat foods (WO)	-	^27^
	Cereus sinensis polysaccharide (CSP-1)	-	^28^
Heavy metals	Lead (Pb); Cadmium (Cd)	-	^29–31^
	Arsenium (As)	-	^32^
	Copper (Cu)	-	^33^
	Silver (Ag)	-	^34^
	Mercury (Hg)	**+**	^35,36^
Natural products	Millet shell polyphenols (MSPs)	**+**	^37^
	Polyphenol-rich plant extract (TOTUM-63)	**+**	^38^
	Green tea polyphenols (GTP)	**+**	^39^
	Qingzhuan tea (QZT)	-	^40^
	Capsaicin (CAP)	**+**	^41^
	Beta-patchoulene (β-PAE)	**+**	^42^
	Macleaya cordata extract (MCE)	**+**	^43^
	Cranberry pomace (CBP)	**+**	^44^
	Blueberry malvidin-3-galactoside (M3G)	**+**	^45^
	Polysaccharide from Pueraria lobata (PPL)	**+**	^46^
	Pectil (5%) & Cellulose (5%)	**+**	^47^
	Extensively hydrolyzed casein formula & *Lactobacillus rhamnosus* GG (EHCF & LGG)	**+**	^48^
	Sophora alopecuroides L.-derived alkaloids	-	^49^
	Flaxseed polysaccharides (FSP)	-	^50^
	Wasabi	-	^51^
Pharmacological intervention	Etifoxine	-	^52,53^
	hydroxychloroquine (HCQ)	-	^54^
	Fungicide thiram	-	^55^
	Trifluoromethanesulfonic acid (TFMS)	**+**	^56^
	Propamocarb (fungicide propamocarb)	**+**	^57^
	Immunoglobulin G (IgG)	**+**	^58^
Exercise and diet	High-intensity interval training & linseed oil (HIIT & LO)	**+**	^59^
	Spontaneous physical activity (PA)	**+**	^60^
	Mediterranean diet	**+**	^61^
	Almond	**+**	^62^
	High-fat diet	**+**	^51^
	High-fat diet	-	^63^
	Fasting	-	^64^
Feed, temperature, and altitude	Grazing & oats hay supplement	**+**	^65^
	High feed efficiency	**+**	^66^
	Fresh forage	**+**	^67^
	High altitude	**+**	^68^
	Fermented feed diets	-	^69^
	Heat stress	-	^70^
Age and gender	Female rat model of Rett syndrome	-	^71^
	Male autism spectrum disorder rodent model	**+**	^72^
	Female autism spectrum disorder rodent model	-	^72^
	Older calves	**+**	^73^
	mature specific-pathogen-free chickens	-	^74^
	Mature rhesus macaques	**+**	^75^

### 2.1 Probiotics and prebiotics

In poultry farming, probiotic *Bacillus* is often mixed with feed to achieve the ultimate goal of ensuring individual health and obtaining high-quality meat. Keerqin et al. found that *Bacillus subtilis* 29,784 significantly improved broilers weight gain and enhanced their gut health status, as well as increased the abundance of *Oscillospira* in the intestinal tract.^16^ Liu et al. added *Bacillus subtilis* to the diets of pullets, which was able to improve the growth performance and intestinal function and induce *Oscillospira* to gradually become the dominant species in the gut.^76^ Similarly, during neonatal broiler chicken rearing, the addition of probiotic *bacillus* preparations (*Bacillus amyloliquefaciens* plus *Bacillus subtilis*) increased the *Oscillospira* abundance and significantly reduced the horizontal transmission of pathogenic *E. coli*, and alleviated the severity of the infection.^17^ Probiotics have also performed well in animal models exploring specific disease-related conditions. Zhao et al. found that *Bacillus coagulans* 13002 significantly alleviated cyclophosphamide-induced intestinal damage in mice and substantially increased the abundance of beneficial microbes such as *Oscillospira* in the mouse gut through microbiota modulation.^15^ Tian et al. used *Bifidobacterium breve* ATCC 15700 (BB) to treat mice exposed to chronic alcohol intake, followed by analyzing the parameters of intestinal flora and liver injury, they found that there was a significant negative correlation between alcoholic liver disease (ALD) and *Oscillospira*, while ALD mice treated with BB showed remission of symptoms and a significant increase in *Oscillospira* abundance.^18^ Some probiotic generated proteins and products also have the similar effects. Exopolysaccharide isolated from *Leuconostoc pseudomesenteroides* XG5 (XG5-EPS) significantly increased the richness of mouse cecum microbiota, especially increasing the relative abundance of *Oscillospira* at the genus level and the relative abundance of *Firmicutes* at the phylum level.^19^ The effect of Tibet kefir milk (TKM) that co-fermented with lactic acid bacteria and yeast, on fat deposition in rats fed high-fat diets with human-derived flora-associated (HFA), was investigated, and it was found that TKM reduced abdominal fat deposition and the triglyceride (TG) levels in serum at the transcriptional level by regulating *Lpl* and *Angptl*4 genes, while increasing *Oscillospira* abundance.^20^ However, not all probiotics can directly or indirectly increase *Oscillospira* abundance. Gamallat et al. found that long-term supplementation with *Lactobacillus rhamnosus* reduced female Sprague Dawley Rats body weight, improved serum cytokines, and reduced serum lipoprotein profiles, while their gut *Oscillospira* abundance was significantly downregulated.^21^ Similarly, *Clostridium butyricum* is capable of producing butyric acid, which has been shown to limit lipid deposition in the liver, restore intestinal barrier function, and improve liver inflammation, with probiotic potential.^77^ Liu et al. found that *Clostridium butyricum* was used in the colitis-associated colon cancer mice and it had alleviated the intestinal inflammation and was accompanied by a decrease in the relative abundance of *Oscillospira*.^22^

In addition to probiotics, prebiotics also has an important impact on the gut microbiota.^78^ Zhao et al. found that oligofructose (FOS) significantly increased the abundance of *Oscillospira* in the mouse gut, especially when FOS was combined with probiotics was further able to inhibit many harmful gut microbes.^15^ Fucoidan (FUC) and galactooligosaccharides (GOS) improved serum dyslipidemia, bile salt hydrolase (BSH) activity, and bile acid-related metabolic levels and promoted *Oscillospira* abundance in the gut of rats with a high-fat diet; meanwhile, *in vitro* tests it revealed that FUC and GOS stimulated BSH activity in *Lactobacillus casei* DM 8121.^23^ Human milk oligosaccharides are very important and have a unique and diverse structure, which can influence the development and composition of the gut microbiota of infants and children through different mechanisms.^79^ A human mouse model of infant fecal transplantation was used to study the effects of fucosyl-α1,3-GlcNAc (3FN), fucosyl-α1,6-GlcNAc, lacto-N-bioside (LNB), and galacto-N-bioside on fecal microbiota and host-bacteria interactions, and it was found that all these disaccharides significantly upregulate *Oscillospira* abundance.^24^ Li et al. found that pea fiber improved the health status in overweight individuals and increased their intestinal *Oscillospira* abundance, meanwhile, they found that the increased *Oscillospira* was significantly associated with the decreased deoxycholic acid (DCA) and lithocholic acid (iso-LCA) in the stool.^25^ Potato fiber is a by-product of starch production and is rich in dietary fiber such as pectin, cellulose, hemicellulose, and resistant starch, which can be utilized and metabolized by gut microbiota to produce SCFAs.^80^ One of the representatives, FiberBind 400, is a commercially available potato fiber product.^26^ In the Gastro-Intestinal Model (TIM)-2 colon model assay, the ingestion of FiberBind 400 increased the intestinal survival of exogenous *Lactobacillus fermentum* PCC®, *Lactobacillus rhamnosus* LGG®, *Lactobacillus reuteri* RC-14®, *Lactobacillus paracasei* F-19® and was also found to promote the growth of intestinal *Oscillospira* by cross-feeding effect.^26^ However, there were also some prebiotics and substances with prebiotic-like properties that were negatively correlated with the abundance of *Oscillospira*. Zhu et al. found that oat β-glucan (OG), oat resistant starch (ORS), and whole oat foods (WO) significantly improved symptoms in the type II diabetic rats and reduced their gut *Oscillospira* abundance.^27^ Cui et al. found that the marine-animal-derived *Cereus sinensis* polysaccharide (CSP-1) significantly increased thymus, spleen index, and total SCFAs production in mice and decreased *Oscillospira* abundance, and they hypothesized that CSP-1 might be a potential prebiotic.^28^

Taken together, probiotics and prebiotics typically exhibit health-promoting effects in poultry farming as well as in specific disease-related animal models, where the vast majority of probiotics or prebiotics intake can directly or indirectly increase the abundance of *Oscillospira* in the host intestinal, whereas, a small number do the opposite, the up-or down-regulation of *Oscillospira* abundance appears to be associated with specific strains or specific prebiotics.

### 2.2 Heavy metal

Studies on the effects of heavy metals on the gut microbiota and host health represent a significant portion of the overall intestinal microbiota-related research.^81^ There is growing epidemiological evidence revealed that heavy metals may contribute to and influence the progression of various metabolic diseases whose etiology and progression are due, in part, to heavy metal-induced disorders of the gut microbiota.^82,83^ By studying the effects of different doses of lead (Pb) exposure on the gut microbiota and gut barrier in mice, Yu et al. found that with increasing doses of Pb, damage to mouse colonic tissue increased, while the relative abundance of *Coprococcus* and *Oscillospira* in the gut decreased linearly, while *Lactobacillus* increased, and Pb exposure had significant effects on the three genera in a dose-dependent manner.^29^ It has been shown that Pb and cadmium (Cd) exposure can affect the concentration of SCFAs by altering the abundance of related microbes that produce SCFAs, such as *Ruminococcus, Bacteroides, Oscillospira*.^30^ Several studies have shown that *Oscillospira* produced butyrate and propionate, increased cupped cell and mucus production, maintained the integrity of the intestinal epithelium, and reduced Pb absorption, thereby reducing colonic tissue damage and inflammation.^5,84–87^ Chi et al. found that 4-week-old C57BL/6 female mice ingested drinking water containing 100 ppb arsenic (As) for 13 weeks showed a significant decrease in the *Oscillospira* abundance, but this was accompanied by an increase in *Akkermansia* and *Bifidobacterium* abundance.^32^ Similarly, Gao et al. found a significant decrease in the abundance of *Oscillospira* in the gut of female mice that ingested drinking water containing 10 ppm Pb for 13 weeks,^31^ a phenomenon consistent with those results of the experiment by Yu et al.^29^ In addition, *Oscillospira* appears to be equally sensitive to other heavy metals, such as copper and silver, which can significantly affect the proportion of *Oscillospira* in the gut of rats.^33,34^ However, there were also heavy metals that positively correlated with *Oscillospira* abundance, such as Hg exposure that caused intestinal damage in mice and increased their intestinal *Oscillospira* abundance,^35^ and this phenomenon was further confirmed in another study.^36^ Notably, it has been shown that higher concentrations of lead, arsenic, copper, zinc, mercury, calcium, and magnesium in the pediatric autism spectrum disorder (ASD) population, especially As and Hg concentrations, were associated with intestinal *Oscillospira* abundance was highly correlated.^88^

The effect of different heavy metals on the abundance of *Oscillospira* in the gut varies. The up-or down-regulation of *Oscillospira* abundance alone does not seem to indicate whether a specific heavy metal has a positive or negative effect on the host health. Therefore, the patterns of *Oscillospira* abundance under different heavy metal exposures need to be further explored, and this genus may also have the potential to be used as one of the indicators for assessing the degree of heavy metal contamination.

### 2.3 Natural products

The effect of natural products on the intestinal microbiota has been studied more frequently; among them, polyphenols seem to be able to play an important role on the host microbiota.^89^ Millet shell polyphenols (MSPs) extracted from the cereal husks have anti-atherosclerotic effects *in vitro*.^90–92^ Liu et al. used ApoE-/- mice fed a high-fat diet as experimental subjects to investigate the anti-atherosclerotic activity of MSPs *in vivo* and found that MSPs effectively inhibited the development of atherosclerotic plaques, reduced the levels of related inflammatory factors, and significantly upregulated the expression levels of tight junction proteins (ocludin, zona occludens-1 and claudin 1) at the mRNA and protein levels; also, MSPs significantly altered the structure of mouse gut microbiota, in which *Oscillospira* and *Ruminococcus* were significantly enriched.^37^ TOTUM-63 (T-63) is a polyphenol-rich plant extract that can have beneficial effects on the body weight and the insulin resistance in mice on a high-fat diet (HFD).^38^ The combination of high-intensity interval training (HIIT) and T-63 was applied to a Western diet-induced obesity rat model, and it was found that this combined modality significantly limited the weight gain and improved the blood glucose levels in the rats, while their *Oscillospira* abundance was also significantly increased.^38^ Green tea polyphenols (GTP) can also improve the abundance of *Oscillospira* in the gut of female sprague-dawley rats.^39^ However, polyphenols were also not all positively correlated with *Oscillospira* abundance. It has been shown that Qingzhuan tea (QZT) has significant anti-obesity, free radical scavenging, antioxidant, inhibiting the proliferation of 3 T3-L1 preadipocytes and other health effects.^93,94^ Feng et al. found that QZT extract ameliorated gut microbiota-mediated metabolic disorders in the high-fat mice and reduced the abundance of *Oscillospira*, which was significantly positively associated with the metabolic syndrome.^40^ In addition to polyphenols, some other active products have similar effects. Capsaicin (CAP) is an active ingredient in chili peppers with a variety of pharmacological activities and potential effects on psychiatric disorders.^95–97^ CAP was found to improve depression and serum levels of 5-hydroxytryptamine (5-HT) and tumor necrosis factor-α (TNF-α) in mice with lipopolysaccharide (LPS)-induced depressive-like behavior and to significantly upregulate the relative abundance of key microbes such as *Oscillospira*.^41^ Liu et al. found that *Oscillospira* became the dominant genus in the gut of β-patchoulene (β-PAE)-treated mice with colitis.^42^ Macleaya cordata contains many important alkaloids, including sanguinarine, chelerythrine, protopine, allocryptopine, and phenolic acids.^98^ The addition of Macleaya cordata extract (MCE) as a dietary supplement to pig feed improved their growth performance and reduced the diarrhea score.^99,100^ Li et al. found that MCE intake significantly increased the relative abundance of *Oscillospira* in the jejunum of weaned pigs.^43^ Cranberry pomace (CBP) is rich in polyphenols, complex carbohydrates, fiber and nutritional minerals.^101^ Continuous addition of CBP during the rearing of broiler chickens eventually significantly increased the abundance of *Oscillospira* in their gut.^44^ Blueberry malvidin-3-galactoside (Blueberry M3G) also increased the gut microbial diversity in mice and significantly increased the abundance of *Oscillospira* and *Ruminococcus*.^45^ Chen et al. found that polysaccharide was derived from pueraria lobata (PPL) not only reduced the isovaleric acid concentrations in the normal mice, but also significantly increased *Oscillospira* abundance and ultimately alleviated antibiotic-associated diarrhea (AAD) induced colonic pathological changes and dysbiosis of intestinal flora in mice.^46^ Gut microbiota also plays an important role in improving cognition and shaping behavior.^102,103^ Mailing et al. found that 5% pectin mixed with 5% cellulose improved mice learning and memory and significantly increased their gut *Oscillospira* abundance.^47^ Likewise, gut microbiota plays a crucial role in food allergies.^104^ In a study of changes in the gut microbiota in patients with wheat-dependent exercise-induced anaphylaxis (WDEIA), *Oscillospira* was positively associated with the ω-5 alcohol-soluble protein-specific immunoglobulin E (IgE), whereas *Bifidobacterium* was significantly negatively correlated with the total IgE levels.^105^ Another study found that *Oscillospira* was highly enriched in the gut of milk-tolerant infants compared to children who remained allergic after treatment with extensively hydrolyzed casein formula plus *Lactobacillus rhamnosus* GG (EHCF + LGG).^48^ However, there was a negative correlation between some natural products and *Oscillospira* abundance. Zhang et al. found that *Sophora alopecuroides* (Leguminosae) L.-derived alkaloids improved depression-like behaviors and depression-related indicators in the chronic unpredictable mild stress (CUMS)-induced depression model mice and decreased *Oscillospira* abundance.^49^ Yang et al. found that the high-fat-diet-fed mice treated with flaxseed polysaccharide (FSP) had significantly decreased their serum fasting glucose, total triglyceride and total cholesterol levels and significantly increased the proportion of beneficial *Akkermansia* and *Bifidobacterium* ratio, while decreasing the proportion of *Oscillospira*.^50^ Thomaz et al. found that wasabi powder significantly improved the health status of diet-induced obese rats and down-regulated their intestinal *Oscillospira* abundance.^51^

Taken together, the natural products represented by polyphenols were able to significantly improve the health status of animal models for specific diseases, particularly metabolic diseases such as obesity caused by high-fat diets, and were accompanied by a significant upregulation of intestinal *Oscillospira* abundance. In addition, other natural active products showed beneficial effects on host health in general, but the abundance of *Oscillospira* under the action of different products varied considerably, and more studies are needed to explore these relationships.

### 2.4 Pharmacological interventions

Pharmacological interventions have an important impact on the gut microbiota.^106,107^ Age-related macular degeneration (AMD) is the main cause of visual impairment in the elderly. Treatment of AMD mice with etifoxine significantly reduced the abundance of *Oscillospira* in the gut.^52^ Etifoxine is also a therapeutic agent for obesity, which was found to down-regulate the relative abundance of *Oscillospira* in the obese mouse model.^53^ Hydroxychloroquine (HCQ) is a widely used antimalarial drug that is recommended for the treatment of coronavirus disease 2019 (COVID-19).^108^ Short-term high-dose HCQ stimulation in mice altered the structure of their gut microbiota, in particular the abundance of *Oscillospira*, but did not affect their intestinal integrity and immune response.^54^ Kong et al. found that fungicide thiram disrupted the gut microbiota of chickens, causing disruption of lipid metabolism and significantly reducing the abundance of *Oscillospira*.^55^ However, the intervention of some drugs can increase the relative abundance of *Oscillospira*. For example, trifluoromethanesulfonic acid (TFMS)-treated mice showed increased abundance of *Oscillospira*.^56^ Wu et al. consistently treated C57BL/6 J mice with fungicide propamocarb and found that their bile acid metabolism was disturbed and *Oscillospira* abundance was increased.^57^ Liu et al. found a significant positive correlation between immunoglobulin G (IgG) and *Oscillospira* abundance in the gut of male Brandt’s voles (*Lasiopodomys brandtii*), with a concomitant increase in *Oscillospira* abundance with increasing IgG concentration.^58^ During the pharmacological interventions, the gut and gut microbiota are the central sites of drug metabolism and drug efficacy, and the metabolic process of different drugs may have the participation of different gut microbes, while the variation of *Oscillospira* abundance may also vary from drug to drug.^109^

### 2.5 Exercise and diet

Dietary structure and exercise patterns have been shown to influence the host health and the gut microbial composition.^110,111^ Plissonneau et al. found that the high-intensity interval training (HIIT) had a significant effect on the gut microbial diversity in Wistar rats and that HIIT alone only had a significant effect on their body fat mass, but when HIIT was combined with linseed oil (LO) improved the conversion of α-linolenic acid (ALA) to docosahexaenoic acid (DHA) and significantly increased the relative abundance of *Oscillospira* in the colonic microbiota.^59^ Notably, neither HIIT nor LO alone resulted in significant changes in the intestinal mucosa-associated flora, but when used in combination, it significantly increased *Oscillospira* abundance. The results of that study were similar to those of previous studies in that *Oscillospira* was negatively correlated with the body mass index (BMI)^112,113^ and positively correlated with leanness.^114^ Similarly, Maillard et al. found a significant increase in the abundance of *Oscillospira* in the gut microbiota of mice after their spontaneous physical activity (PA), accompanied by a significant increase in the level of SCFAs.^60^ Two animal experiments showed a strong correlation between *Oscillospira* abundance, lactate levels, and exercise intensity.^115,116^ In a population-based trial, Haro et al. found that obese people on a Mediterranean diet for one year showed a decrease in the gut *Prevotella* abundance and a significant increase in *Oscillospira* abundance.^61^ Thus, *Oscillospira* is considered as a possible next-generation probiotic candidate for weight loss and fat reduction.^2^ In a randomized controlled trial, short-term consumption of almonds also increased *Oscillospira* abundance in healthy adults.^62^ A high-fat diet has been considered a non-healthy diet. Thomaz et al. found that a high-fat diet increased *Oscillospira* abundance in the gut of rats,^51^ however, the opposite result was seen in another animal test, where Schots et al. found a significant decrease in *Oscillospira* abundance in the gut of female mice on a high-fat diet,^63^ and it is unknown whether this is due to species differences. In addition, He et al. found that fasting reduced the relative abundance of *Oscillospira* in the human intestine,^64^ suggesting that calorie restriction may have a negative regulatory effect on *Oscillospira*. Overall, appropriate exercise patterns and moderate exercise levels appear to increase the relative abundance of *Oscillospira* in both human and animal, while an accepted healthy dietary structure also positively regulates the increase in *Oscillospira* abundance.

### 2.6 Other factors

*Oscillospira* was first discovered in the rumen of animals, and feed and grazing practices have had a wide impact on this intestinal organism. Ahmad et al. found that the combination of grazing and concentrate supplement significantly increased the abundance of *Oscillospira* in the rumen flora of yak (Bos grunniens).^65^ The cecum microbiota plays an important role in the host food digestion and nutrient absorption, and to some extent affects feed efficiency (FE). Notably, *Oscillospira* showed a stronger positive correlation in the high feed efficiency group (HFE) than that in the low feed efficiency group (LFE).^66^ *O. guilliermondii* was detected in the rumen of several herbivorous animals, including cattle and sheep, and the abundance of this organism was significantly higher especially when the diet was fresh forage.^67^ However, in the opposite case, Yan et al. fed geese with fermented feed diets prepared by co-fermentation of *Bacillus, Lactobacillus*, and yeast with corn, soybean meal, and wheat bran and found a significant downregulation of *Oscillospira* abundance.^69^

Heat stress also down-regulated the abundance of *Oscillospira* in the broilers’ intestine.^70^ *Oscillospira* is commonly found in the gut of herbivores in high-altitude environments, accounting for nearly 20% of the total microbiota.^68^ Host characteristics such as sex and age are closely related to the structure and function of the gut microbiota.^117^ Sequencing analysis of rhesus macaques (*Macaca mulatta*) fecal samples revealed that female macaques had higher levels of alpha diversity and a more distinctive microbial structure than males, and those mature individuals had a higher abundance of *Oscillospira* compared to immature individuals.^75^ Similarly, the abundance of *Oscillospira* in male and female ASD rodent models exhibited substantial differences, and these differences may be due to sex specificity.^71,72^ Xi et al. found that the abundance of *Oscillospira* in the gut of specific-pathogen-free chickens decreased with increasing age.^74^ Maron et al. found that *Oscillospira* was commonly found in the gut of older calves compared to younger calves.^73^

## 3. *Oscillospira*-related diseases

In studies of gut microbiota and their related diseases, *Oscillospira* often appears in high-throughput sequencing data, and it is particularly noteworthy that *Oscillospira* abundance fluctuates widely in some specific diseases. Here, we give a summary of diseases positively or negatively associated with *Oscillospira* (Table 2).

**Table 2.** *Oscillospira*-related diseases

Positively	Subject	References	Negatively	Subject	References
High-fat diet-induced obesity (HFDIO)	Mice	^52,118^	Ulcerative colitis (UC)	UC patients	^119^
			Inflammatory bowel disease (IBD)	Children	^120^
High-fat diet-induced type 2 diabetic (T2DM)	Rat	^27^	T2DM	Rat	^121^
DSS-induced ulcerative colitis (UC)	Mice	^122^	Crohn’s disease (CD)	Patient	^123^
			Pediatric nonalcoholic fatty liver disease	Obese patients	^124^
Chronic kidney disease (CKD)	Idiopathic nephrotic syndrome patients	^125^	Chronic inflammation	Geriatric population	^126^
Parkinson’s disease (PD)	PD patients	^127^	PD	PD patients	^128^
Autism spectrum disorder (ASD)	Children	^129^	Overweight and obesity	Children	^130^
Chronic unpredictable mild stress (CUMS)	Mice	^131^	Obesity	Obese patient	^132^
Gallstone	Gallstones patients	^133^	Fragile X syndrome (FXS)	Mice	^134^
Chronic constipation	Female patients	^135^	CUMS	Mice	^49^
			Autism	Children	^136^
			Fatty liver	Adolescents	^137^
			Nonalcoholic fatty liver disease (NAFLD); nonalcoholic steatohepatitis (NASH)	Patient	^138^
			Alcoholic liver disease	Mice	^18^
			Lung cancer	Patient	^139^
			Loose stools	Patient	^140^

### 3.1 Diseases positively associated with oscillospira

Data from animal experiments show that a high-fat diet appears to promote an increase in intestinal *Oscillospira* abundance. Compared to the normal group (NOR), the mice on the high-fat diet-induced obesity and showed dysbiosis of the intestinal flora, with a significantly higher abundance of *Oscillospira*,^118^ a result that was also seen in another high-fat diet mouse test.^52^ *Oscillospira* abundance in the gut of Type 2 diabetes mellitus (T2DM) rats was positively correlated with the development of diabetes and inflammation,^27^ however, in contrast, *Oscillospira* abundance in another trial was extremely low.^121^ In the dextran sulfate sodium (DSS)-induced ulcerative colitis in mice test, DSS was observed to increase the percentage of *Oscillospira* in the gut.^122^ Zhang et al. found that patients with chronic kidney disease (CKD) had a high abundance of *Oscillospira* in the gut.^125^ There is also a close association between central neurological and degenerative disorders and *Oscillospira*. Parkinson’s disease (PD) patients also had a high abundance of *Oscillospira*,^127^ but this result showed the opposite trend in another study.^128^ Zhai et al. sequenced and analyzed the gut microbiota of ASD children and found that *Oscillospira* significantly increased.^129^ In another study of CUMS-induced depression-like mice, CUMS induction resulted in increased abundance of *Oscillospira*, while this genus decreased after Kai-Xin-San (KXS) treatment.^131^ What can be determined is that, *Oscillospira* is directly associated with gallstones and this organism can be used as a biomarker for symptomatic gallstone formation.^133^ Keren et al. found that patients with gallstones had higher total fecal bile acids (BAs) concentrations and lower microbial diversity, accompanied by increased abundance of *Oscillospira*, which was further analyzed and found to be positively correlated with secondary BAs and negatively correlated with primary BAs.^133^ It was worth noting that slow transmission/constipation was a definite risk factor in the formation of gallstones.^141^ It has been shown that fast colonic transit times select for fast-growing microbes, while slower transit/constipation allows slower replicating organisms to remain in the lumen and avoid being eluted.^13^ *Oscillospira* showed high abundance in this case, most likely due to its slow growth and thus benefiting from a slow transit time in the gut. Thus, a high abundance of *Oscillospira* was positively correlated with constipation, especially in the female population with chronic constipation, and this correlation was very strong.^135^

### 3.2 Diseases negatively associated with oscillospira

Currently, several studies have shown that inflammation is strongly associated with *Oscillospira*, and most of them have a negative correlation.^2^ Xu et al. found that *Oscillospira* abundance was negatively correlated with disease severity in patients with ulcerative colitis (UC).^119^ Lima et al. found a lower abundance of *Oscillospira* in the gut of children with inflammatory bowel disease (IBD).^120^ A significantly lower abundance of *Oscillospira* was also found in the gut of Crohn’s disease (CD) patients and pediatric nonalcoholic fatty liver disease (NALD) patients.^123,124^ Aging is a low-grade chronic inflammation characterized by elevated circulating levels of inflammatory mediators.^142^ This chronic inflammation occurs in the absence of obvious infection is called inflammation and is a risk factor for morbidity and mortality in the elderly population.^143^ In addition, the important role of gut microbiota perturbation with aging has been revealed, and a growing body of literature suggests that age-related gut microbiota dysbiosis contributes to the overall inflammatory status of older adults.^144^ Among them, *Oscillospira* showed a strong negative correlation with pro-inflammatory monocyte chemoattractant protein-1 (MCP-1).^126^ Childhood obesity is a global health problem, and gut microbiota plays an extremely important role in obesity.^145^ Chen et al. found a significant decrease in the abundance of *Oscillospira* in the overweight children gut.^130^ Similarly, Verdam et al. found a significant decrease in *Oscillospira* abundance in the gut of obesity-associated diabetic patients, while *O. guillermondii* was significantly decreased in obese patients with local and systemic inflammation.^132^ Fragile X syndrome (FXS) is a neurodevelopmental disorder that is considered to be the most common cause of genetic intellectual disability and one of the major predisposing factors for autism.^146,147^ Altimiras et al. found that *Oscillospira* abundance was significantly downregulated in the gut of FXS mouse models.^134^ A strong correlation between *Oscillospira* and several depression-related indicators was also previously confirmed in another trial.^49^ This relationship appears to be further supported by data from a population-based experiment, where Johnson et al. found that social competence (a composite measure of participants’ extraversion, social skills, and communication ability) was highly positively correlated with *Akkermansia, Lactococcus*, and *Oscillospira*, among them, *Oscillospira* was more abundant in individuals with higher sociality scores.^136^ Tian et al. studied mice exposed to chronic alcohol intake by analyzing gut microbiota and liver injury parameters and found that the development of ALD was accompanied by a significant decrease in *Oscillospira* abundance.^18^ Clinically, *Oscillospira* abundance was also negatively correlated with hepatic fat;^137^ the abundance of *Oscillospira* in the gut of pediatric nonalcoholic fatty liver disease (NAFLD) and nonalcoholic steatohepatitis (NASH) patients was similarly reduced. Currently, reduced *Oscillospira* accompanied by increased 2-butanone has been identified as a gut microbiota signature of NAFLD onset.^138^ Lung cancer pathogenesis was accompanied by a significant decrease in *Oscillospira* abundance, with a negative correlation between the two.^139^ A study of gut microbiota and stool softness/hardness in European adults showed that *Oscillospira* abundance was positively correlated with harder stools and negatively correlated with loose stools.^140^

## 4. Potential applications of *oscillospira*

*Oscillospira* is currently described only in high-throughput sequencing data related to the gut microbiota. Pure cultures of this organism have not been obtained and therefore, the actual biology, function, and specific role of *Oscillospira* in the gut microbiota and human health have not been conclusively established. The current description of *Oscillospira* is mainly reflected in the variation of its abundance in different environments.^3^ Through multiple studies in Table 2, we found that *Oscillospira* is highly associated with obesity and obesity-related chronic inflammatory and metabolic diseases and that *Oscillospira* abundance is significantly decreased in this category of disease. In addition, several studies have confirmed that *Oscillospira* is strongly associated with leanness or lower BMI in children and adults, and shows a high degree of heritability.^112,132,148,149^ Numerous evidences suggest that *Oscillospira* abundance plays an important role in the metabolic activities associated with obesity in humans. *Oscillospira* may be a next-generation probiotic candidate with weight loss, lipid-lowering, slimming, and metabolic syndrome-relieving effects, and it has great potential for health applications. In addition, there is an association between *Oscillospira* and central nervous system disorders and degenerative diseases, but more studies are needed to reveal the underlying mechanisms as the evidence is scarce and the causality has not been confirmed. It is noteworthy that *Oscillospira* is more abundant in people with gallstones and chronic constipation, and it is not known what role this organism play can either foster health or contribute to disease in the development of such diseases. It should be seen that *Oscillospira* plays an important role in the gut microbiota and its abundance is closely related to the host health. External interventions such as probiotics, prebiotics, polyphenols, diet, and exercise can significantly influence the abundance of *Oscillospira* in the gut, which opens up the possibility of targeted interventions for the prevention, mitigation, and treatment of specific diseases mediated by intestinal flora, such as obesity and obesity-related diabetes. In the future, microecological formulations with *Oscillospira* as the core may bring new options for food, nutraceuticals, and biopharmaceuticals for consumption or medicinal use.

## 5. Conclusions

Overall, *Oscillospira* exhibited beneficial microbial traits. In particular, *Oscillospira* directly or indirectly exhibits positive regulatory effects in areas related to obesity and chronic inflammation. Therefore, *Oscillospira* has the potential to be developed as the next generation of probiotics. In the future, more preclinical and clinical studies are needed to confirm the efficacy of *Oscillospira* in different diseases, and until then, if the pure culture technology of this organism can be overcome, then it will greatly accelerate its development and application process.
